# Retrospective analysis of clinical efficacy and safety of fluorometholone combined with azelastine eye drops for seasonal allergic conjunctivitis

**DOI:** 10.3389/fmed.2026.1807309

**Published:** 2026-05-04

**Authors:** Xiangjun Dai, Jun Yu, Li Niu, Xiao-Hua Chen

**Affiliations:** 1 Department of Comprehensive Ophthalmology, Yiwu Aier Eye Hospital, Zhejiang, China; 2 Department of Refractive Surgery, Yiwu Aier Eye Hospital, Zhejiang, China

**Keywords:** azelastine, combined therapy, efficacy, fluorometholone, retrospective analysis, safety, seasonal allergic conjunctivitis

## Abstract

**Background:**

Seasonal allergic conjunctivitis (SAC) is a common ocular allergic disease, predominantly affecting children and young adults, with no significant gender predominance.

**Aim:**

This study aimed to retrospectively evaluate the clinical efficacy and safety of fluorometholone combined with azelastine eye drops versus azelastine monotherapy in patients with SAC.

**Methods:**

This single-center retrospective study enrolled 180 SAC patients from January 2022 to June 2024. Patients were divided into a combination group (fluorometholone 0.1% QID + azelastine 0.05% BID, *n* = 90) and a monotherapy group (azelastine 0.05% BID, *n* = 90). Symptom and sign scores were assessed at baseline, weeks 1, 2, 4, and 2 months post-treatment. Efficacy, recurrence rate, and adverse events were compared.

**Results:**

The combination group showed significantly greater reduction in symptom and sign scores from week 1 onward (*p* < 0.01). At week 2, the total effective rate was 98.89% in the combination group versus 81.11% in the monotherapy group (*p* < 0.05). The recurrence rate at 2 months post-treatment was significantly lower in the combination group (6.7% vs. 17.8%, χ^2^ = 4.889, *p* = 0.027). Adverse events were mild and comparable between groups (5.6% vs. 4.4%, *p* = 0.697).

**Conclusion:**

Fluorometholone combined with azelastine is more effective than azelastine monotherapy in treating SAC, providing faster symptom relief, higher efficacy, lower recurrence, and a comparable safety profile.

## 1 Introduction

Seasonal allergic conjunctivitis (SAC) is the most common subtype of allergic conjunctivitis and accounts for the majority of ocular allergic diseases worldwide (approximately 80–90%) (1–3). It commonly affects children and young adults, although it can occur at any age, with no consistent gender predominance (1, 2). The prevalence of SAC is higher in regions with increased exposure to airborne allergens, particularly pollen, and is influenced by geographic and environmental factors (2, 4). SAC is an IgE-mediated inflammatory disorder of the conjunctiva triggered by environmental allergens such as pollen, dust mites, and mold spores (1, 3). Clinically, it is characterized by seasonal exacerbations, typically occurring during periods of high pollen exposure, such as spring and early autumn (1, 4). Clinical manifestations include ocular pruritus, conjunctival hyperemia, lacrimation, foreign body sensation, and edema, which can significantly impair patients’ quality of life and, in severe cases, lead to corneal epithelial damage (5, 6). The pathogenesis of SAC follows a classic type I hypersensitivity reaction, which can be divided into sensitization and effector phases. During the sensitization phase, initial exposure to airborne allergens (e.g., pollen) is processed by antigen-presenting cells and presented to T helper 2 (Th2) cells, leading to B-cell class switching to IgE production. These allergen-specific IgE antibodies bind with high affinity to FcεRI receptors on the surface of conjunctival mast cells, thereby “priming” the mast cells for subsequent responses. In the effector phase, re-exposure to the same allergen cross-links the surface-bound IgE on these primed mast cells, triggering rapid degranulation and the release of preformed mediators (e.g., histamine, tryptase) and *de novo* synthesized mediators (e.g., leukotrienes, prostaglandins, cytokines). These mediators induce vasodilation, increased vascular permeability, recruitment of inflammatory cells (eosinophils, neutrophils), and activation of sensory nerves, resulting in the typical clinical manifestations of itching, hyperemia, chemosis, and tearing (7, 8). The released mediators orchestrate both the early-phase and late-phase allergic responses. The early phase occurs within minutes of allergen re-exposure and is primarily driven by preformed mediators, particularly histamine, which directly induces vasodilation, increased vascular permeability, and stimulation of sensory nerve endings, resulting in acute ocular itching, hyperemia, and tearing. Tryptase and chymase further amplify the response by activating protease-activated receptors and promoting plasma extravasation. The late phase develops 4–8 h later and is sustained by *de novo* synthesized mediators, including leukotrienes (e.g., LTB4, LTD4), prostaglandins (e.g., PGD2), and pro-inflammatory cytokines (e.g., IL-4, IL-5, IL-13, TNF-*α*). These mediators promote the recruitment and activation of eosinophils, neutrophils, and Th2 lymphocytes, leading to persistent chemosis, palpebral conjunctival papillae formation, and epithelial damage. This biphasic pattern explains the prolonged and recurrent nature of clinical signs and symptoms in SAC (8, 9).

First-line treatment often involves antihistamine eye drops for rapid symptom relief. Azelastine hydrochloride (e.g., Optivar), a second-generation antihistamine, exerts its therapeutic effects through dual mechanisms: it acts as a selective histamine H1 receptor antagonist, blocking the downstream activation of phospholipase C and calcium mobilization, thereby rapidly alleviating itching and hyperemia; additionally, it stabilizes mast cell membranes by inhibiting calcium influx and modulating intracellular signaling pathways, thereby reducing the release of preformed and newly synthesized inflammatory mediators such as histamine, leukotrienes, and tryptase (9, 10). However, its efficacy in controlling moderate-to-severe inflammatory signs (e.g., significant hyperemia, edema) can be limited, and recurrence rates post-treatment may be high (11).

Topical glucocorticoids are potent anti-inflammatory agents. Fluorometholone (e.g., FML), a weak-to-moderate potency corticosteroid, exerts its anti-inflammatory effects primarily through binding to cytoplasmic glucocorticoid receptors (GR-*α*), leading to nuclear translocation and subsequent transactivation of anti-inflammatory genes (e.g., annexin A1, IL-10) and transrepression of pro-inflammatory transcription factors such as nuclear factor-κB (NF-κB) and activator protein-1 (AP-1). This results in downregulation of pro-inflammatory cytokines (e.g., IL-4, IL-13, TNF-*α*, eotaxin), inhibition of inflammatory cell recruitment, and stabilization of vascular endothelial cells. This mechanism offers effective local inflammation control with a lower risk of intraocular pressure elevation compared to stronger steroids, making it suitable for short-term use in allergic conjunctivitis (12–15). Theoretically, this combination could provide synergistic benefits, addressing both immediate symptoms and underlying inflammation (16, 17). However, robust clinical data for this specific combination in SAC, particularly regarding recurrence rates from real-world settings, are limited. Therefore, this retrospective study aimed to compare the clinical efficacy, safety, and recurrence rate of fluorometholone combined with azelastine versus azelastine monotherapy in SAC patients treated at our hospital.

## 2 Materials and methods

### 2.1 Study design and patients

This single-center, retrospective observational study was conducted at the Ophthalmology Outpatient Department of our hospital from January 2022 to June 2024. The study protocol was approved by the Hospital Ethics Committee (Approval No.: 202201006) and adhered to the Declaration of Helsinki. Informed consent was waived due to the retrospective nature, but patient data were anonymized and confidentiality maintained.

Inclusion Criteria: (1) Diagnosis of SAC per the Chinese Ophthalmological Society guidelines (18) and consistent with international criteria (e.g., EAACI) (10), with symptoms correlating to seasonal allergen exposure; (2) Age 18–65 years; (3) New diagnosis or history of SAC but no acute attack in the preceding 3 months; (4) Received one of the two study regimens and completed 4-week treatment plus 2-month follow-up; (5) Complete medical records. Exclusion Criteria: (1) Other conjunctivitis types (bacterial, viral, vernal keratoconjunctivitis); (2) Co-existing ocular diseases (e.g., active corneal disease, glaucoma, uveitis) affecting assessment; (3) Known hypersensitivity to study drugs; (4) Use of other anti-allergic/anti-inflammatory ophthalmic drugs within 1 week prior; (5) Severe uncontrolled systemic diseases (e.g., hypertension, diabetes, autoimmune disorders); (6) Pregnancy or lactation; (7) Lost to follow-up.

Disease severity was assessed at baseline based on the total symptom score (TSS) and total sign score (TgSS). Patients were classified as mild (TSS + TgSS < 8), moderate (8–14), or severe (> 14) according to the diagnostic and grading criteria established by the Corneal Disease Group of the Chinese Ophthalmological Society (19, 20). Baseline severity distribution was compared between groups to ensure balance. Exploratory subgroup analyses were performed to evaluate whether the treatment effect differed by baseline disease severity; however, these analyses were not pre-specified and should be interpreted with caution due to the limited sample size within each severity subgroup.

### 2.2 Grouping and intervention

A total of 180 eligible patients were enrolled. According to the treatment regimen, they were divided into a combination therapy group (90 cases, 90 eyes) and a monotherapy group (90 cases, 90 eyes). Combined Treatment Group (n = 90): Received fluorometholone eye drops (0.1%, 1 drop/eye, QID) + azelastine hydrochloride eye drops (0.05%, 1 drop/eye, BID) for 4 weeks. Drops were administered at least 15 min apart. Monotherapy Group (n = 90): Received azelastine hydrochloride eye drops alone (0.05%, 1 drop/eye, BID) for 4 weeks. All patients were advised on allergen avoidance and ocular hygiene.

### 2.3 Outcome measures

Assessments occurred at baseline, weeks 1, 2, 4, and 2 months post-treatment.

(1) Symptom and Sign Scoring: According to the diagnostic criteria for allergic conjunctivitis established by the Corneal Disease Group of the Chinese Ophthalmological Society (19, 20), the TSS was calculated as the sum of scores for ocular itching, lacrimation, and foreign body sensation (each 0–3, maximum 9). The TgSS was calculated as the sum of scores for conjunctival hyperemia and chemosis, palpebral conjunctival papillae, and discharge (each 0–3, maximum 9). Detailed scoring criteria are as follows:① Ocular itching: 1 point (mild itching), 2 points (moderate, tolerable itching), 3 points (severe, intolerable itching). ② Lacrimation: 1 point (increased tear production without outflow), 2 points (mild tearing), 3 points (frequent tearing, accompanied by clear nasal discharge). ③ Foreign body sensation: 1 point (mild, tolerable, no pain), 2 points (accompanied by tearing, no pain), 3 points (severe, accompanied by pain). ④ Conjunctival hyperemia and chemosis: 1 point (mild conjunctival vascular dilation), 2 points (moderate, between mild and severe), 3 points (marked conjunctival vascular dilation, obscuring vascular pattern). ⑤ Palpebral conjunctival papillae: 1 point (involving <1/3 of the palpebral conjunctival surface), 2 points (involving 1/3 to 1/2 of the surface), 3 points (involving >1/2 of the surface). ⑥ Discharge: 1 point (small amount of sticky, mucous, watery, or ropy discharge), 2 points (moderate amount, requiring eye wiping 3–4 times daily), 3 points (large amount of discharge, requiring eye wiping more than 5 times daily). The detailed scoring criteria for symptoms and signs are summarized in Table 1.

**Table 1:** Scoring criteria for ocular symptoms and signs in seasonal allergic conjunctivitis.

Item	Score 0	Score 1	Score 2	Score 3
Symptoms				
Ocular pruritus	None	Mild, tolerable	Moderate	Severe, intolerable
Lacrimation	None	Increased, no outflow	Mild tearing	Frequent tearing with rhinorrhea
Foreign body sensation	None	Mild, tolerable	Moderate with tearing	Severe with pain
Signs				
Conjunctival hyperemia and chemosis	None	Mild vasodilation	Moderate	Marked, obscured vascular pattern
Palpebral conjunctival papillae	None	<1/3 surface	1/3–1/2 surface	> 1/2 surface
Discharge	None	Small amount (watery/ropey/mucous)	Moderate (3–4 wipes/day)	Abundant (> 5 wipes/day)
Severity grading	Total score (TSS + TgSS)			
Mild	—	<8	—	—
Moderate	—	8–14	—	—
Severe	—	>14	—	—

TSS, total symptom score (range 0–9); TgSS, total sign score (range 0–9). Scoring system was established by the corneal disease group of the Chinese ophthalmological society.

(2) Efficacy Evaluation Criteria: ① Cured: Complete resolution of all ocular symptoms and signs. ② Effective: Improvement in ocular symptoms and signs compared with pre-treatment. ③ Ineffective: No improvement or worsening of ocular symptoms and signs. Total effective rate = (Cured cases + Effective cases) / Total number of cases × 100%.

(3) Safety Assessment: Adverse events were monitored, including elevated intraocular pressure (IOP), drug-induced glaucoma, drug-induced cataract, and fungal infection. IOP was measured using non-contact tonometry at baseline and at each follow-up visit (weeks 1, 2, and 4). IOP elevation was defined as an increase of ≥ 5 mmHg from baseline or IOP > 21 mmHg. All IOP measurements were performed by trained ophthalmic technicians prior to administration of eye drops on the day of assessment to avoid transient fluctuations induced by topical medication.

All assessments were performed by two trained ophthalmologists who were not involved in treatment allocation. However, due to the retrospective nature of this study, the evaluators were not masked to the treatment groups, which may introduce potential observer bias. To minimize this bias, standardized scoring protocols were strictly followed, and inter-observer consistency was maintained through regular calibration.

### 2.4 Statistical analysis

Data were analyzed using SPSS 26.0 (IBM Corp., Armonk, NY, USA). Continuous data were expressed as mean ± standard deviation (SD) and compared using independent t-tests or Mann–Whitney U tests based on normality assessed by the Shapiro–Wilk test. Paired t-tests or Wilcoxon signed-rank tests were used for within-group comparisons. Categorical data were expressed as frequencies (n, %) and compared using the chi-square (χ^2^) test or Fisher’s exact test, as appropriate. Changes in TSS and TgSS over time were analyzed via repeated-measures analysis of variance (ANOVA) with Greenhouse–Geisser correction for sphericity, examining time, group, and interaction effects. For exploratory subgroup analyses by baseline disease severity (mild, moderate, severe), descriptive comparisons were performed; however, no formal interaction tests were conducted due to limited sample sizes, and results should be interpreted as hypothesis-generating rather than confirmatory. A two-sided *p* value < 0.05 was considered statistically significant. No adjustment for multiple comparisons was applied, given the exploratory nature of the subgroup analyses.

## 3 Results

### 3.1 Baseline characteristics and overall treatment effects

A total of 180 patients were enrolled in this study, including 90 patients in the combined treatment group and 90 patients in the monotherapy group. All patients completed the 4-week treatment and 2-month follow-up, and no patients were lost to follow-up or withdrew from the study due to adverse reactions.

The baseline characteristics of the two groups, including age, gender, disease duration, history of allergic rhinitis, baseline TSS, TgSS, and total score (TSS + TgSS), were comparable (all *p* > 0.05). Baseline disease severity distribution (mild/moderate/severe: 22/57/11 vs. 21/58/11) was also balanced between groups (χ^2^ = 0.086, *p* = 0.958), indicating good comparability (Table 2).

**Table 2:** Comparison of baseline characteristics between the two groups of patients.

Characteristic	Combined treatment group (*n* = 90)	Monotherapy group (*n* = 90)	t/χ^2^	*P*
Age (years)	38.6 ± 10.2	37.9 ± 9.8	0.423	0.673
Gender, *n* (%)			0.125	0.724
Male	42 (46.7)	44 (48.9)	-	-
Female	48 (53.3)	46 (51.1)	-	-
Disease duration (days), mean ± SD	7.8 ± 3.2	8.1 ± 3.5	0.571	0.569
History of allergic rhinitis, *n* (%)	52 (57.8)	55 (61.1)	0.221	0.638
Baseline scores				
Total symptom score (TSS, 0–9)	6.1 ± 1.8	6.3 ± 1.9	0.721	0.472
Total sign score (TgSS, 0–9)	4.2 ± 1.5	4.4 ± 1.6	0.845	0.399
Total score (TSS + TgSS, 0–18)	10.3 ± 2.9	10.7 ± 3.1	0.894	0.372

**Figure 1:**
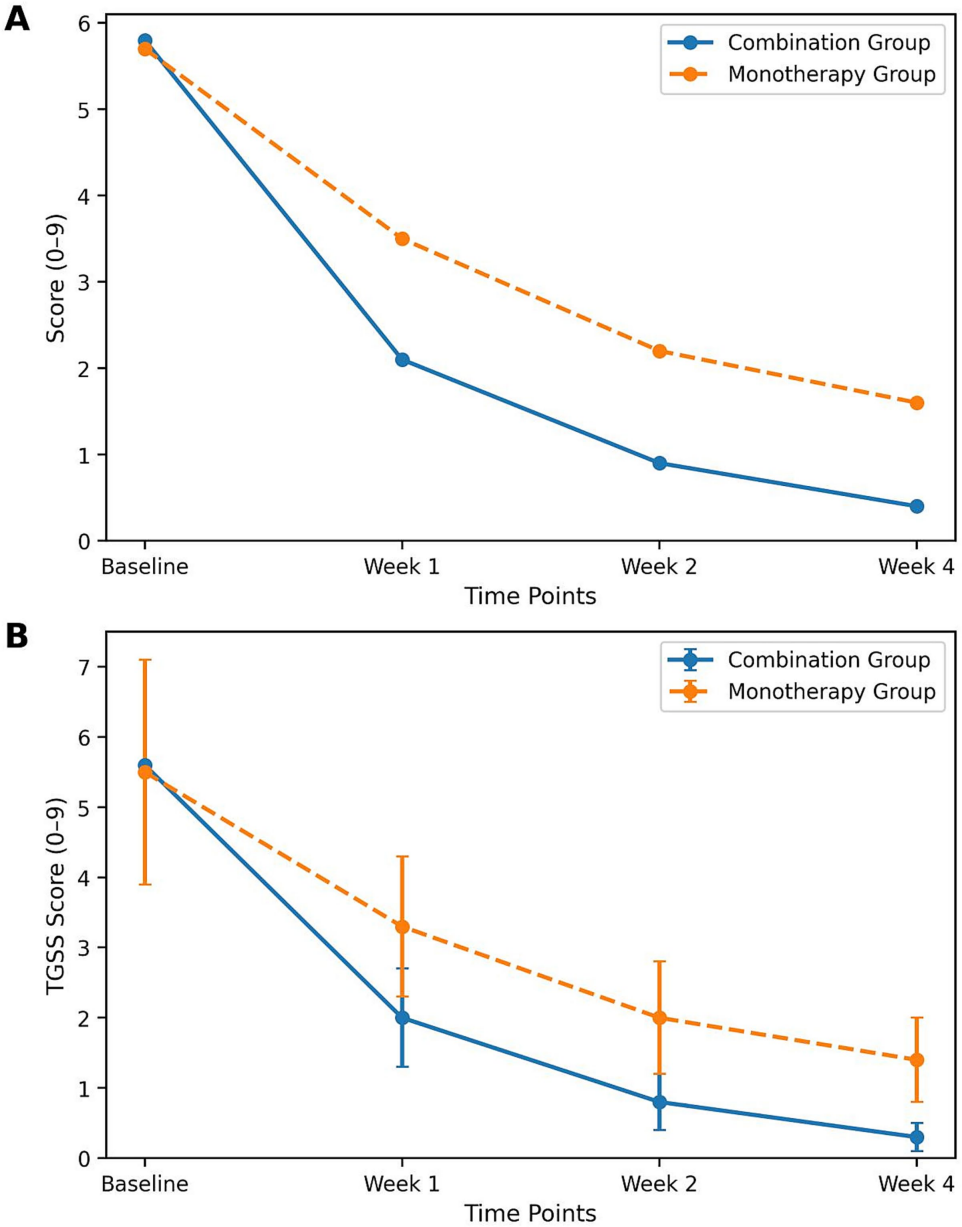
Mean changes in total symptom score (TSS) and total sign score (TgSS) from baseline to week 4. **(A)** Changes in total symptom score (TSS) from baseline to week 4 note: mean changes in total symptom score (TSS) from baseline to week 4 in the combination group and monotherapy group. Data are presented as mean ± standard deviation (SD). The combination group showed a significantly greater and faster reduction in TSS compared with the monotherapy group at all post-treatment time points (week 1, week 2, week 4; all *p* < 0.001). **(B)** Changes in total sign score (TgSS) from baseline to week 4 in the combination and monotherapy groups. Data are presented as mean ± SD. The combination group showed a more pronounced reduction in TgSS compared with the monotherapy group over time.

Repeated-measures ANOVA for TSS revealed significant main effects of time (*F* = 328.41, df = 1.76, 312.37, *p* < 0.001), group (*F* = 116.75, df = 1, 178, *p* < 0.001), and time × group interaction (*F* = 67.32, df = 1.76, 312.37, *p* < 0.001). Repeated-measures ANOVA for TgSS also showed significant main effects of time (*F* = 296.53, df = 1.82, 323.24, *p* < 0.001), group (*F* = 98.46, df = 1, 178, *p* < 0.001), and time × group interaction (*F* = 52.19, df = 1.82, 323.24, *p* < 0.001). These results confirmed that the combination group exhibited a significantly faster and greater reduction in symptoms and signs over time compared with the monotherapy group (Table 3). The changes in total symptom score (TSS) over time are illustrated in Figure 1.

**Table 3:** Summary of efficacy outcomes at different time points in the two groups (mean ± SD).

Outcome	Group	Baseline	Week 1	Week 2	Week 4
Total symptom score (TSS)	Combination	6.24 ± 0.51	3.87 ± 0.57ab	3.03 ± 0.42ab	2.53 ± 0.38ab
	Monotherapy	5.99 ± 0.72	5.35 ± 0.61a	3.96 ± 0.45a	3.87 ± 0.62a
Total sign score (TgSS)	Combination	5.21 ± 1.18	3.66 ± 1.22ab	2.80 ± 1.15ab	1.93 ± 0.98ab
	Monotherapy	5.02 ± 1.25	4.47 ± 1.38a	3.69 ± 1.29a	3.02 ± 1.11a
Total score (TSS + TgSS)	Combination	10.3 ± 2.9	7.53 ± 1.68ab	5.83 ± 1.41ab	4.46 ± 1.16ab
	Monotherapy	10.7 ± 3.1	9.82 ± 2.45a	7.65 ± 1.83a	6.89 ± 1.65a
Total effective rate, %	Combination	—	—	98.89ab	—
	Monotherapy	—	—	81.11	—

aP < 0.01 vs. baseline within the same group; bP < 0.01 vs. monotherapy group at the same time point.

Exploratory subgroup analyses based on baseline disease severity suggested that combination therapy was associated with greater improvements in TSS and TgSS from week 1 onward in both moderate and severe subgroups, whereas in mild cases, both treatments achieved satisfactory outcomes with faster symptom relief observed in the combination group. However, given the small sample sizes within each severity subgroup, these findings are preliminary and require confirmation in larger prospective studies.

### 3.2 Comparison of symptom scores

Before treatment, there were no significant differences in individual symptom scores (ocular itching, lacrimation, foreign body sensation) or TSS between the two groups (all *p* > 0.05).

Compared with baseline, both groups showed significant reductions in all individual symptom scores and TSS at 1, 2, and 4 weeks (all *p* < 0.001). At all post-treatment time points, ocular itching, foreign body sensation, and TSS were significantly lower in the combination group than in the monotherapy group (all *p* < 0.001). Lacrimation score was significantly lower in the combination group at 1 week (*p* < 0.001) and 4 weeks (*p* < 0.001), with no significant difference at 2 weeks (*p* = 0.072) (Table 4).

**Table 4:** Comparison of symptom scores between the two groups before and after treatment (mean ± SD, points).

Group	Symptom	Baseline	1 week	2 weeks	4 weeks
Monotherapy group (*n* = 90)	Ocular itching	2.19 ± 0.30	1.89 ± 0.53a	1.36 ± 0.34a	1.34 ± 0.57a
	Lacrimation	1.96 ± 0.69	1.82 ± 0.27	1.34 ± 0.58a	1.30 ± 0.40a
	Foreign body sensation	1.84 ± 0.34	1.64 ± 0.35 a	1.26 ± 0.36a	1.23 ± 0.73 a
	Total symptom score	5.99 ± 0.72	5.35 ± 0.61a	3.96 ± 0.45a	3.87 ± 0.62a
Combined treatment group (*n* = 90)	Ocular itching	2.29 ± 0.48	1.20 ± 0.33ab	1.01 ± 0.27ab	0.90 ± 0.25ab
	Lacrimation	2.04 ± 0.36	1.35 ± 0.69ab	1.16 ± 0.72	0.92 ± 0.33ab
	Foreign body sensation	1.91 ± 0.19	1.32 ± 0.36ab	0.86 ± 0.41ab	0.71 ± 0.20ab
	Total symptom score	6.24 ± 0.51	3.87 ± 0.57ab	3.03 ± 0.42ab	2.53 ± 0.38ab

aP < 0.01 vs. baseline within the same group; bP < 0.01 vs. monotherapy group at the same time point.

### 3.3 Comparison of sign scores

Baseline individual sign scores (bulbar conjunctival congestion and edema, palpebral conjunctival papillae, discharge) and TgSS were comparable between groups (all *p* > 0.05).

Within-group comparisons showed significant reductions in all sign scores and TgSS at 1, 2, and 4 weeks in both groups (all *p* < 0.001). At all post-treatment time points, bulbar conjunctival congestion and edema, discharge, and TgSS were significantly lower in the combination group (all *p* < 0.001). Palpebral conjunctival papillae score was significantly lower in the combination group at 4 weeks (*p* < 0.001), with no significant differences at 1 week (*p* = 0.124) and 2 weeks (*p* = 0.068) (Table 5).

**Table 5:** Comparison of individual sign scores and total sign score between the two groups before and after treatment (mean ± SD, points).

Group	Sign	Baseline	1 week	2 weeks	4 weeks
Monotherapy group (*n* = 90)	Bulbar conjunctival congestion and edema	2.21 ± 0.41	1.85 ± 0.52a	1.42 ± 0.48a	1.05 ± 0.39a
	Palpebral conjunctival papillae	1.52 ± 0.56	1.48 ± 0.55	1.32 ± 0.51a	1.21 ± 0.47a
	Discharge	1.29 ± 0.63	1.14 ± 0.58	0.95 ± 0.52a	0.76 ± 0.45a
	Total sign score	5.02 ± 1.25	4.47 ± 1.38a	3.69 ± 1.29a	3.02 ± 1.11a
Combined treatment group (*n* = 90)	Bulbar conjunctival congestion and edema	2.25 ± 0.39	1.52 ± 0.49ab	1.01 ± 0.42ab	0.58 ± 0.35ab
	Palpebral conjunctival palpebral conjunctival papillae	1.61 ± 0.53	1.38 ± 0.50a	1.18 ± 0.48a	0.82 ± 0.41ab
	Discharge	1.35 ± 0.60	0.76 ± 0.48ab	0.61 ± 0.43ab	0.53 ± 0.40ab
	Total sign score	5.21 ± 1.18	3.66 ± 1.22ab	2.80 ± 1.15ab	1.93 ± 0.98ab

aP < 0.01 vs. baseline within the same group; bP < 0.01 vs. monotherapy group at the same time point.

### 3.4 Comparison of total effective rate

After 4 weeks of treatment, the total effective rate was 98.89% in the combination group and 81.11% in the monotherapy group. The difference was statistically significant (χ^2^ = 15.120, *p* < 0.001) (Table 6).

**Table 6:** Comparison of therapeutic effects between the two groups [*n* (%)].

Group	*n*	Cured	Effective	Ineffective	Total effective rate (%)
Monotherapy group (*n* = 90)	90	43 (47.78)	30 (33.33)	17 (18.89)	81.11
Combined treatment group (*n* = 90)	90	58 (64.44)	31 (34.44)	1 (1.11)	98.89a

Compared with the control group, the difference in the total effective rate was statistically significant (aP < 0.001).

### 3.5 Comparison of recurrence rate

At 2 months after treatment, the recurrence rate was 6.7% (6/90) in the combination group and 17.8% (16/90) in the monotherapy group. The combination group had a significantly lower recurrence rate (χ^2^ = 4.889, *p* = 0.027) (Table 7).

**Table 7:** Comparison of recurrence rate between the two groups at 2 months after treatment.

Group	*n*	Number of relapses(*n*)	Recurrence rate (%)	*p*
Monotherapy group	90	16	17.8	0.027
Combined treatment group	90	6	6.7	-

### 3.6 Safety evaluation

Adverse events were mild and self-limited in both groups, including transient ocular burning, dryness, and foreign body sensation. No significant IOP elevation, cataract, or severe complications occurred.

The overall incidence of adverse events was 5.6% (5/90) in the combination group and 4.4% (4/90) in the monotherapy group, with no significant difference (χ^2^ = 0.152, *p* = 0.697) (Table 8).

**Table 8:** Comparison of adverse reactions between the two groups [*n* (%)].

Adverse reactions	Combined treatment group (*n* = 90), *n* (%)	Monotherapy group (*n* = 90), *n* (%)	χ^2^ value	*P* value
Ocular burning	3 (3.3)	2 (2.2)	-	-
Ocular dryness	1 (1.1)	1 (1.1)	-	-
Foreign body sensation	1 (1.1)	1 (1.1)	-	-
Total adverse reactions	5 (5.6)	4 (4.4)	0.152	0.697

*n* (%) = frequency (percentage); No moderate or severe adverse reactions occurred in either.

Mean IOP remained stable throughout treatment in both groups (all *p* > 0.05), with no clinically significant IOP elevation observed.

## 4 Discussion

This retrospective study demonstrates that for patients with active SAC, a 4-week course of fluorometholone combined with azelastine eye drops is superior to azelastine monotherapy in accelerating symptom and sign resolution, achieving a higher clinical response rate, and reducing short-term recurrence without increasing adverse events.

The superior efficacy of the combination aligns with the synergistic pharmacologic rationale. Azelastine provides rapid relief by blocking histamine H1 receptors and stabilizing mast cells. Fluorometholone, through glucocorticoid receptor-mediated actions, exerts broad anti-inflammatory effects by suppressing pro-inflammatory cytokine transcription (e.g., IL-4, IL-13, TNF-*α*), inhibiting inflammatory cell recruitment, and stabilizing vascular endothelial cells (21, 22). This dual attack likely explains the significantly faster and greater reduction in both subjective symptoms and objective inflammatory signs observed from week 1 onward (23, 24).

The significantly lower 2-month recurrence rate observed in the combination group warrants further mechanistic consideration. Beyond the immediate anti-inflammatory effects, fluorometholone may contribute to sustained remission through several pathways. First, by suppressing the late-phase allergic inflammatory cascade via NF-κB and AP-1 inhibition, it reduces the infiltration and persistence of eosinophils, which are key drivers of chronic allergic inflammation. Second, chronic inflammation is known to impair ocular surface epithelial barrier function by disrupting tight junction proteins such as occludin and zonula occludens-1 (ZO-1). The anti-inflammatory action of fluorometholone may facilitate restoration of ocular surface epithelial barrier integrity, thereby reducing susceptibility to allergen re-exposure. Third, emerging evidence suggests that topical corticosteroids may modulate local immunological memory by reducing the recruitment and activation of memory T cells in the conjunctiva, although this hypothesis requires further investigation. Collectively, these mechanisms may explain why the combination regimen, which addresses both the early-phase mast cell-driven response and the late-phase inflammatory cascade, achieves more durable symptom control compared with antihistamine monotherapy (25).

Safety is a paramount concern with corticosteroid use. The absence of significant intraocular pressure elevation or other steroid-related complications in our cohort is reassuring and consistent with the known safety profile of short-term (≤4 weeks) use of weak-to-moderate potency steroids like fluorometholone (26, 27). The similar incidence of mild, self-limiting local adverse events between groups underscores the favorable safety of this combination regimen. The mild adverse events observed in both groups, including ocular burning, dryness, and foreign body sensation, are consistent with the known tolerability profiles of topical antihistamines and corticosteroids. Notably, ocular dryness may be attributed in part to the anticholinergic properties of azelastine, which exhibits weak muscarinic receptor antagonist activity. Blockade of M3 receptors on lacrimal glands can reduce tear secretion, contributing to transient ocular surface dryness. This effect is typically dose-dependent and self-limiting, and no patient in our cohort required discontinuation due to dry eye symptoms. The similar incidence of adverse events between the combination and monotherapy groups suggests that the addition of fluorometholone does not exacerbate local tolerability concerns (28–30).

## 5 Limitations and future directions

This study has several limitations inherent to its retrospective, non-randomized, single-center design. First, treatment allocation was based on physician judgment rather than randomization, introducing potential selection bias. Although baseline characteristics were comparable between groups, unmeasured confounding factors—such as disease severity, prior treatment history, or patient adherence—may have influenced outcomes. Second, the lack of objective biomarkers (e.g., conjunctival eosinophil count, tear cytokine levels) limits the mechanistic depth of our findings. Third, the follow-up period was relatively short for assessing long-term safety and recurrence patterns (31, 32). SAC is a seasonal disease, and a 2-month post-treatment follow-up may not capture recurrences occurring during the next pollen season. Future studies should extend follow-up to at least 6 months or through a full seasonal cycle to more accurately evaluate long-term efficacy and recurrence rates. Fourth, the exploratory subgroup analyses by disease severity were limited by small sample sizes within each subgroup and were not pre-specified; therefore, these findings should be considered hypothesis-generating and require validation in larger, adequately powered prospective trials. Fifth, while we monitored intraocular pressure at each follow-up visit, the 4-week treatment duration may not be sufficient to detect late-onset steroid-related complications such as cataract or chronic IOP elevation. Long-term safety studies are warranted (33, 34).

Future prospective, randomized, multi-center trials with larger sample sizes, longer follow-up, and objective inflammatory biomarkers are needed to confirm these findings. Research should incorporate objective inflammatory biomarkers and evaluate the optimal duration of combination therapy (35). Comparisons with other combination regimens (e.g., antihistamine + nonsteroidal anti-inflammatory drug, dual-action anti-allergic agents) would also be valuable.

## 6 Conclusion

In this retrospective analysis, fluorometholone combined with azelastine eye drops was more effective than azelastine monotherapy in the treatment of SAC, resulting in faster and more complete resolution of clinical manifestations and a lower short-term recurrence rate, without increasing adverse events. This combination represents a viable and effective therapeutic strategy for managing active SAC in clinical practice.

## Data Availability

The original contributions presented in the study are included in the article/supplementary material, further inquiries can be directed to the corresponding author.
